# Lyrical Content of Contemporary Popular Music (1999-2018) and the Role of Healthcare Providers in Media Education of Children and Adolescents

**DOI:** 10.7759/cureus.11214

**Published:** 2020-10-28

**Authors:** Elise Kury, Erin Kury, Nolan Quinn, Robert P Olympia

**Affiliations:** 1 Pediatrics, University of New England College of Osteopathic Medicine, Biddeford, USA; 2 Pediatrics, Pennsylvania College of Health Sciences, Lancaster, USA; 3 Pediatrics, Elizabethtown College, Elizabethtown, USA; 4 Emergency Medicine, Penn State Hershey Medical Center, Hershey, USA

**Keywords:** music, lyrics, media education, violence, sexual behavior, substance use

## Abstract

Music can affect the behavior and emotion of children and adolescents. We conducted a content and trend analysis of Billboard’s top 10 songs, 1999-2018, with particular attention to adult and positive themes. There was a total of 3633 total references, 3298 (90.8%) adult themes, and 335 (9.2%) positive themes. The most common adult themes were “sexually suggestive lyrics” (32.2%), “sexually suggestive dancing” (15.6%), “use of swear words” (13.7%), “alcohol use” (4.7%), and “use of guns/deadly weapons” (4.7%). The most common positive theme was “empowerment” (62.3%). There were greater references per minute for adult themes (4.8 vs. 3.7 references per minute) and positive themes (0.6 vs. 0.3 references per minute) in the 2009-2018 study period as compared with the 1999-2008 study period. We encourage pediatric healthcare providers to be active participants in the promotion of media education, and we encourage parents to be mindful of the level of media exposure.

## Introduction

Music is an important aspect in the socialization of children and adolescents, often providing entertainment, frameworks for self-identity and the acquisition of morals, distraction from stressors, simple cures for boredom or loneliness, and structure for the development of relationships [1-4].

Studies have shown that children and adolescents listen to music an average of 40 hours per week and anywhere from one to 6.8 hours per day [5-7]. The exposure of children and adolescents to music can occur in many different ways. Contemporary popular music can be heard in the background at restaurants or places of recreation or business, pop-up or television commercials, films or television shows, video games, concerts (live or live-streaming), or social media (Facebook, Twitter, Instagram, TikTok, etc.).

Access to music is readily available (purchasing albums or compact discs have been replaced with downloadable or streaming music on portable handheld electronic devices or “Wi-Fi” home devices) and free of cost (such as Pandora, Spotify, or YouTube). Oftentimes, a child or adolescents’ choice of music is private due to the use of headphones or lack of parental supervision. Although there are parental locks for most electronic devices, tech-savvy children and adolescents are finding ways around them. In addition, while many social media and streaming services have “restriction mode” to block mature content, it requires the parent/guardian to be familiar with and activate the option. While radio stations and music streaming radio services often “censor” songs, what words/lyrics are deleted or modified is dependent on the specific station and not always regulated. When searching for popular songs on the internet, the “clean” censored version will appear; unfortunately, these versions may only censor specific vulgar words and not general adult themes or mature overtones.

Several studies have examined the content and effect of music on children and adolescents and have concluded that exposure to music can affect behavior and emotion [8] and can increase risk-taking behaviors such as aggression/violence [9-10], suicide [11-13], use of tobacco, alcohol, and illicit drugs [14-16], and unsafe sexual practices [17-18].

The effect of music is based on the age of the child or adolescent, the emotional and developmental stage of the listener, the level of exposure, and the genre of music [1]. Media education has been shown to reduce the harmful effects of media and strengthen the positive effects [19-20]. Media education enables the child or adolescent to limit his or her use of media, to make positive media choices, to select alternatives to media consumption, and to develop critical thinking and viewing skills [1].

There have been several studies examining the lyrical content of music from a variety of genres. These studies have focused on risk-taking behaviors such as substance use/abuse [21-24], violence [25-26], and degrading and non-degrading sexual behaviors [27]. There have been no published studies looking at the lyrical content of contemporary popular music from a variety of genres over two decades, focusing on both adult themes and positive themes. The objective of our study was to perform content and trend analysis of contemporary popular music released from 1999 to 2018, with particular attention to adult themes and positive themes.

## Materials and methods

We conducted a content analysis study during the summer of 2019, examining adult and positive themes found in a select number of popular songs. We analyzed the top 10 songs identified by Billboard’s year-end “Hot 100 Songs” (https://www.billboard.com/charts/year-end) for each year between 1999 and 2018, for a total of 200 songs. Billboard rankings of the “Hot 100 Songs” were based on national radio airplay, streaming online data, digital sales data, and YouTube views. Data abstracted for each song included the year of release, artist or artists, genre (Popular (Pop), Hip Hop/Rap, Rhythm & Blues (R&B)/Soul, Rock, Country, House, Alternative/Indie, and Electronic); several songs were categorized with more than one genre)), and song length (minute/seconds).

A data collection instrument was developed by the co-investigators, composed of a predetermined list of adult and positive themes, each theme defined prior to data collection. Adult themes included violent crime (physical assault, sexual assault, murder, self-harm, and use of guns/deadly weapons), non-violent crime (theft, gambling, the glorification of prison, gang glorification, and vandalism), use of inappropriate language (use of swear words, racism, homophobia, and sexual harassment), sexuality (sexually suggestive lyrics, performing sexual acts, infidelity, and prostitution), substance use (alcohol use, selling drugs, drug use), and partying (sexually suggestive dancing, partying/clubbing). Positive themes included positive body image, empowerment, camaraderie, and positive romantic relationships.

Written lyrics were accessed for each of the 200 songs from Lyric Find (https://lyricfind.com/). The three reviewers (E.K, E.K., and N.Q) collectively completed the data collection instrument for each song, noting each time that an adult and positive theme was stated or implied (defined as a “reference”). Each reference could be included in more than one themed category based on the reviewers’ collective interpretation of the lyrics.

The total number of references for each of the adult and positive themes were determined for all 200 songs in the sample and then determined for each of the 20 years during the study period, as well as stratified into two study periods: 1999-2008 and 2009-2018. The percentages, with 95% confidence intervals, of major adult and positive themes were calculated for the entire study period (1999-2018) and then for each of the two study periods, 1999-2008 and 2009-2018. A t-test was used to compare the means of two groups of continuous data. In addition, the number of references per minute for major adult and positive themes was determined for all 200 songs in the sample, for each of the 20 years during the study sample, and for the two stratified study periods (1999-2008 and 2009-2018).

The Institutional Review Board at the Pennsylvania State Hershey Medical Center deemed the study exempt.

## Results

Analysis was performed on 200 songs. (Table 1) The most common genres associated with our sample of songs was Pop (42.2%), R&B/Soul (17.2%), and Hip hop/Rap (16.0%). (Table 2) 

**Table 1:** Demographics of selected songs for analysis (1999-2018)

Year of release	Song title	Song artist	Genre(s)	Song length in minutes
1999	Believe	Cher	Pop	3:59
1999	No Scrubs	TLC	R&B	4:00
1999	Angel of Mine	Monica	R&B/Soul	4:10
1999	Heartbreak Hotel	Whitney Houston	Rock	4:40
1999	Baby One More Time	Britney Spears	Pop	3:31
1999	Kiss Me	Sixpence None The Richer	Pop	3:31
1999	Genie In A Bottle	Christina Aguilera	Pop	3:40
1999	Every Morning	Sugar Ray	Rock	3:39
1999	Nobody's Supposed To Be Here	Deborah Cox	R&B Pop	4:22
1999	Livin La Vida Loca	Ricky Martin	Pop	4:03
2000	Breath	Faith Hill	House	4:09
2000	Smooth	Santana ft. Rob Thomas	Rock Pop	4:56
2000	Maria Maria	Santana ft. The Product GandB	Rock Pop	4:23
2000	I Wanna Know	Joe	Rock Pop	4:56
2000	Everything You Want	Vertical Horizon	Alternative/Indie Pop	4:17
2000	Say My Name	Destiny’s Child	Electronic	4:31
2000	I Knew I Loved You	Savage Garden	Rap	4:10
2000	Amazed	Lonestar	Country	4:29
2000	Bent	Matchbox Twenty	Electronic	4:16
2000	He Wasn’t Man Enough	Toni Braxton	R&B Pop	4:21
2001	Hanging By A Moment	Lifehouse	Rock	3:36
2001	Fallin	Alicia Keys	R&B/Soul	3:30
2001	All For You	Janet Jackson	Rock	6:31
2001	Drops Of Jupiter	Train	Pop Rock	4:20
2001	I’m Real	Jennifer Lopez ft. Ja Rule	Pop Hip Hop/Rap	4:58
2001	If You’re Gone	Matchbox Twenty	Rock	4:34
2001	Let Me Blow Ya Mind	Eve ft. Gwen Stefani	Hip Hop/Rap	3:51
2001	Thank You	Dido	Pop	3:44
2001	Again	Lenny Kravitz	Rock	3:50
2001	Independent Women Part 1	Destiny’s Child	Pop	3:37
2002	How You Remind Me	Nickelback	Rock	3:43
2002	Foolish	Ashanti	R&B	3:47
2002	Hot In Herre	Nelly	Pop	3:49
2002	Dilemma	Nelly ft. Kelly Rowland	Hip Hop/R&B	4:49
2002	Wherever You Will Go	The Calling	Rock	3:29
2002	A Thousand Miles	Vanessa Carlton	Pop Rock	3:57
2002	In The End	Linkin Park	Rock	3:36
2002	What’s Luv	Fat Joe ft. Ashanti	Hip Hop/Rap	4:52
2002	U Got It Bad	Usher	R&B/Soul	4:07
2002	Blurry	Puddle of Mudd	Rock	5:04
2003	In Da Club	50 cent	Hip Hop/Rap	3:13
2003	Ignition	R. Kelly	R&B	3:16
2003	Get Busy	Sean Paul	Hip Hop	3:32
2003	Crazy In Love	Beyonce ft. Jay-Z	R&B Pop	4:56
2003	When I’m Gone	3 Doors Down	Hip Hop/Rap	4:20
2003	Unwell	Matchbox Twenty	Rock	3:48
2003	Right Thurr	Chingy	Hip Hop/Rap	4:10
2003	Miss You	Aaliyah	Rock	4:05
2003	Picture	Kid Rock ft. Sheryl Crow	Country	4:58
2003	Bring Me To Life	Evanescence ft. Paul McCoy	Rock	3:56
2004	Yeah!	Usher ft. Lil Jon and Ludacris	Hip Hop/R&B	4:10
2004	Burn	Usher	R&B	4:15
2004	If I Ain’t Got You	Alicia Keys	R&B/Soul	3:48
2004	This Love	Maroon 5	Pop	4:25
2004	The Way You Move	OutKast ft. Sleepy Brown	Hip Hop/Rap	3:55
2004	The Reason	Hoobastank	Rock	3:53
2004	I Don’t Wanna Know	Mario Winans ft. Enya and P.Diddy	R&B/Soul	4:17
2004	Hey Ya	Outkast	Hip Hop/Rap	3:55
2004	Goodies	Ciara ft. Petey Pablo	R&B	3:43
2004	Lean Back	Terror Squad	Hip Hop/Rap	4:07
2005	We Belong Together	Mariah Carey	R&B Pop	3:21
2005	Hollaback Girl	Gwen Stefani	Pop	3:19
2005	Let Me love You	Mario	Electronic	4:09
2005	Since U Been Gone	Kelly Clarkson	Pop	3:08
2005	1, 2 Step	Ciara ft. Missy Elliot	R&B	3:22
2005	Gold Digger	Kanye West ft. Jamie Foxx	Hip Hop/Rap	3:28
2005	Boulevard of Broken Dreams	Green Day	Alternative	4:21
2005	Candy Shop	50 Cent ft. Olivia	Hip Hop/Rap	3:29
2005	Don’t Cha	The Pussycat Dolls ft. Busta Rhymes	Pop	4:32
2005	Behind These Hazel Eyes	Kelly Clarkson	Pop Rock	3:19
2006	Bad Day	Daniel Powter	Pop	3:54
2006	Temperature	Sean Paul	R&B/Soul	3:36
2006	Promiscuous	Nelly Furtado ft. Timbaland	Pop	4:02
2006	You’re Beautiful	James Blunt	Pop	3:32
2006	Hips Don’t Lie	Shakira ft. Wyclef Jean	Pop	3:38
2006	Unwritten	Natasha Bedingfield	Pop	4:18
2006	Crazy	Gnarls Barkley	R&B/Soul Pop	2:58
2006	Ridin’	Charmillionaire ft. Krayzie Bone	Hip Hop/Rap	5:02
2006	Sexyback	Justin Timberlake	Pop	4:02
2006	Check On It	Beyonce ft. Slim Thug	R&B/Soul Pop	3:30
2007	Irreplaceable	Beyoncé	R&B/Soul Pop	3:47
2007	Umbrella	Rihanna ft. Jay-Z	Pop	4:46
2007	The Sweet Escape	Gwen Stefani ft. Akon	Pop	4:06
2007	Big Girls Don’t Cry	Fergie	R&B/Soul Pop	4:28
2007	Buy U A Drank (Shawty Snappin’)	T-Pain ft. Yung Joc	R&B/Soul	3:48
2007	Before He Cheats	Carrie Underwood	Country	3:19
2007	Hey There Delilah	Plain White T’s	Alternative	3:52
2007	I Wanna Love You	Akon ft. Snoop Dogg	Hip Hop/Rap R&B/Soul	4:07
2007	Say It Right	Nelly Furtado	Pop	3:43
2007	Glamorous	Fergie ft. Ludacris	Hip Hop Pop	4:06
2008	Low	Flo Rida ft. T-Pain	Hip Hop/Rap	3:50
2008	Bleeding Love	Leona Lewis	Pop	4:22
2008	No One	Alicia Keys	R&B/Soul Rock Pop	4:10
2008	Lollipop	Lil Wayne ft. Static Major	Hip Hop/Rap	5:03
2008	Apologize	Timbaland ft. OneRepublic	Pop Hip Hop/Rap	3:04
2008	No Air	Jordin Sparks and Chris Brown	Pop	4:23
2008	Love Song	Sara Bareilles	Pop	4:18
2008	Love In This Club	Usher ft. Young Jeezy	R&B	4:19
2008	With You	Chris Brown	Pop	4:12
2008	Forever	Chris Brown	Dance	4:38
2009	Boom Boom Pow	The Black Eyed Peas	Pop	5:08
2009	Poker Face	Lady Gaga	Pop Rock	3:58
2009	Just Dance	Lady Gaga ft. Colby O’Donis	Pop Rock	4:01
2009	I Gotta Feeling	The Black Eyed Peas	Pop	4:50
2009	Love Story	Taylor Swift	Country Pop	3:57
2009	Right Round	Flo Rida	Hip Hop/Rap	3:27
2009	I’m Yours	Jason Mraz	Pop	4:03
2009	Single Ladies (Put A Ring On It)	Beyoncé	R&B Pop	3:13
2009	Heartless	Kanye West	Pop R&B Hip Hop/Rap	3:31
2009	Gives You Hell	The All-American Rejects	Alternative/Indie	3:33
2010	Tik Tok	Ke$ha	Pop	3:20
2010	Need You Now	Lady Antebellum	Country	4:37
2010	Hey, Soul Sister	Train	Pop Rock	3:37
2010	California Gurls	Katy Perry ft. Snoop Dogg	Pop Rock	3:56
2010	OMG	Usher ft. will.i.am	Pop	4:28
2010	Airplanes	B.O.B ft. Hayley Willams	Hip Hop/Rap	3:01
2010	Love The Way You Lie	Eminem ft. Rihanna	Hip Hop/Rap	4:23
2010	Bad Romance	Lady Gaga	Pop Rock	4:54
2010	Dynamite	Taio Cruz	Pop	3:22
2010	Break Your Heart	Taio Cruz ft. Ludacris	Pop	3:23
2011	Rolling In The Deep	Adele	Pop	3:48
2011	Party Rock Anthem	LMFAO ft. Lauren Bennett and GoonRock	Pop	4:23
2011	Firework	Katy Perry	Pop Rock	3:47
2011	E.T.	Katy Perry ft. Kanye West	Pop Rock	3:51
2011	Give Me Everything	Pitbull ft. Ne-Yo, Afrojack and Nayer	Hip Hop/Rap	4:12
2011	Grenade	Bruno Mars	Pop	3:42
2011	Fuck You	Cee Lo Green	Pop	3:41
2011	Super Bass	Nicki Minaj	Pop Hip Hop/Rap	3:30
2011	Moves Like Jagger	Maroon 5 ft. Christina Aguilera	Electronic Pop	3:21
2011	Just Can’t Get Enough	The Black Eyed Peas	Pop	3:39
2012	Somebody That I Used To Know	Gotye ft. Kimbra	Alternative/Indie Pop	4:04
2012	Call Me Maybe	Carly Rae Jepsen	Pop	3:13
2012	We Are Young	Fun. ft. Janelle Monae	Alternative/Indie Pop	4:10
2012	Payphone	Maroon 5 ft. Wiz Khalifa	Pop Electronic	3:51
2012	Lights	Ellie Goulding	Pop Electronic	3:32
2012	Glad You Came	The Wanted	Pop	3:18
2012	Stronger (What Doesn’t Kill You)	Kelly Clarkson	Pop	3:42
2012	We Found Love	Rihanna ft. Calvin Harris	Electronic Pop	3:35
2012	Starships	Nicki Minaj	R&B Hip Hop/Rap	3:30
2012	What Makes You Beautiful	One Direction	Pop	3:18
2013	Thrift Shop	Macklemore and Ryan Lewis ft. Wanz	Hip Hip/Rap	3:55
2013	Blurred Lines	Robin Thicke ft. T.I. and Pharrell	R&B Pop	4:25
2013	Radioactive	Imagine Dragons	Electronic Rock	3:05
2013	Harlem Shake	Baauer	Electronic	3:16
2013	Can’t Hold Us	Macklemore and Ryan Lewis ft. Ray Dalton	Pop	4:18
2013	Mirrors	Justin Timberlake	Pop R&B	8:05
2013	Just Give Me a Reason	P!nk ft. Nate Ruess	Pop	4:02
2013	When I Was Your Man	Bruno Mars	Pop	3:34
2013	Cruise	Florida Georgia Line ft. Nelly	Country	3:29
2013	Roar	Katy Perry	Pop	3:42
2014	Happy	Pharrell Williams	Pop	3:53
2014	Dark Horse	Katy Perry ft. Juicy J	Pop	3:35
2014	All of Me	John Legend	R&B/Soul	4:29
2014	Fancy	Iggy Azalea ft. Charli XCX	House Pop	3:19
2014	Counting Stars	OneRepublic	Pop	4:19
2014	Talk Dirty	Jason Derulo ft. 2 Chainz	Pop	2:58
2014	Rude	Magic!	Pop	3:44
2014	All About That Bass	Meghan Trainor	Pop	3:08
2014	Problem	Ariana Grande ft. Iggy Azalea	Pop R&B	3:14
2014	Stay With Me	Sam Smith	Soul Pop	2:52
2015	Uptown Funk!	Mark Ronson ft. Bruno Mars	R&B	4:30
2015	Thinking Out Loud	Ed Sheeran	Soul Pop	4:41
2015	See You Again	Wiz Khalifa ft. Charlie Puth	Hip Hop/Rap	3:49
2015	Trap Queen	Fetty Wap	Hip Hop/Rap	3:35
2015	Sugar	Maroon 5	Electronic Pop	3:55
2015	Shut Up And Dance	Walk The Moon	Alternative/Indie	3:17
2015	Blank Space	Taylor Swift	Electronic Pop	3:51
2015	Watch Me	Silento	House Electronic R&B/Soul Pop	3:05
2015	Earned It	The Weekend	Pop	4:10
2015	The Hills	The Weekend	R&B	4:02
2016	Love Yourself	Justin Bieber	R&B Pop	3:53
2016	Sorry	Justin Bieber	R&B Pop	3:20
2016	One Dance	Drake ft. WixKid and Kyla	Hip Hop/Rap	2:54
2016	Work	Rihanna ft. Drake	R&B	3:39
2016	Stressed Out	Twenty One Pilots	Rock Hip Hip/Rap	3:22
2016	Panda	Designer	Hip Hop/Rap	4:06
2016	Hello	Adele	Pop	4:55
2016	Don’t Let Me Down	Chainsmokers ft. Daya	Pop Electronic	3:28
2016	Can’t Stop The Feeling	Justin Timberlake	Pop	3:56
2016	Closer	Chainsmokers ft. Halsey	Electronic Pop	4:04
2017	Shape Of You	Ed Sheeran	Pop	3:53
2017	Despacito	Luis Fonsi and Daddy Yankee ft. Justin Bieber	Pop	3:47
2017	That’s What I Like	Bruno Mars	R&B/Soul Hip hop	3:26
2017	Humble	Kendrick Lamar	Hip Hop/Rap	2:57
2017	Something Just Like This	Coldplay and The Chainsmokers	Electronic	4:08
2017	Bad and Boujee	Lil Uzi Vert	Hip Hop/Rap	5:43
2017	Closer	The Chainsmokers ft. Halsey	Electronic Pop	4:04
2017	Body Like A Back Road	Sam Hunt	Country	2:40
2017	Believer	Imagine Dragons	Pop Rock Alternative/Indie	3:24
2017	Congratulations	Post Malone	Hip Hop/Rap	3:40
2018	God’s Plan	Drake	Pop Rap	3:18
2018	Perfect	Ed Sheeran	Pop	4:23
2018	Meant To Be	Bebe Rexha and Florida Georgia Line	Country Pop R&B	2:43
2018	Havana	Camila Cabello ft. Young Thug	Pop	3:36
2018	Rockstar	Post Malone ft. 21 Savage	R&B Hip Hop/Rap	3:38
2018	Psycho	Post Malone ft. Ty Dolla Sign	R&B Hip Hop/Rap	3:40
2018	I Like It	Cardi B, Bunny, and J Balvin	Hip Hop/Rap	4:13
2018	The Middle	Zedd, Maren Morris, and Grey	House Pop Electronic	3:04
2018	In My Feelings	Drake	Hip Hop/Rap	3:37
2018	Girls Like You	Maroon 5 ft. Cardi B	Pop Rock R&B	3:55

**Table 2:** Genres (n=268) represented in our sample of 200 songs (several songs represented more than one genre)

Genre represented	Number of songs (%)
Pop	113 (42.2%)
R&B/Soul	46 (17.2%)
Hip Hop/Rap	43 (16.0%)
Rock	30 (11.2%)
Electronic	17 (6.3%)
Country	8 (3.0%)
Alternative/Indie	7 (2.6%)
House	4 (1.5%)

There was a total of 3633 total references in our sample of 200 songs, 3298 (90.8%) adult themes and 335 (9.2%) positive themes (Table 3). When comparing adult and positive themes, there were greater references per minute for adult themes (4.2 references per minute) compared with positive themes (0.4 references per minute), and this difference was statistically significant (p<0.0001, mean difference 3.8, 95% confidence interval [2.5-5.0]) (Table 3).

**Table 3:** Total references and references per minute for adult and positive themes in our sample of 200 songs, then stratified by 1999-2008 and 2009-2018. Major themes (in italics) presented as total number (percentage [95% confidence interval])

		1999-2018 (n=200 songs, 788.0 minutes)	1999-2008 (n=100 songs, 405.8 minutes)	2009-2018 (n=100 songs, 382.2 minutes)
Violent crime		236 (7.2% [6.3-8.0])	92 (6.2% [5.1-7.6])	144 (7.9% [6.7-9.3])
	Physical assault	48	28	20
	Sexual assault	0	0	0
	Murder	8	0	8
	Self-harm	25	1	24
	Use of guns/deadly weapons	155	63	92
Nonviolent crime		147 (4.5% [3.8-5.2])	31 (2.1% [1.5-3.0])	116 (6.4% [5.3-7.6])
	Theft	33	8	25
	Gambling	44	0	44
	Glorification of Prison	5	2	3
	Gang glorification	34	8	26
	Vandalism	31	13	18
Use of inappropriate language		661 (20.0% [18.7-21.4])	226 (15.3% [13.5-17.2])	435 (23.9% [22.0-25.9])
	Use of swear words	453	142	311
	Racism	88	49	39
	Homophobia	4	3	1
	Sexual harassment	116	32	84
Sexuality		1202 (36.4% [34.8-38.1])	672 (45.5% [42.9-48.0])	530 (29.1% [27.1-31.3])
	Sexually suggestive lyrics	1062	571	491
	Performing sexual acts	77	42	35
	Infidelity	60	58	2
	Prostitution	3	1	2
Substance use		354 (10.7% [9.7-11.8])	83 (5.6% [4.6-6.9])	271 (14.9% [13.3-16.6])
	Alcohol use	155	59	96
	Selling drugs	78	3	75
	Drug use	121	21	100
Partying		698 (21.2% [19.9-22.6])	374 (25.3% [23.2-27.6])	324 (17.8% [16.1-20.0])
	Sexually suggestive dancing	514	248	266
	Partying/clubbing	184	126	58
Total references -adult themes (references per minute)		3298 (4.2 references per minute)	1478 (3.7 references per minute)	1820 (4.8 references per minute)
Positive themes				
	Positive body image	28 (8.3% [5.8-11.6])	11 (9.0% [5.0-15.6])	17 (8.0% [5.0-12.5])
	Empowerment	210 (62.3% [57.4-67.7])	93 (76.2% [68.0-83.0]	117 (54.9% [48.2-61.5])
	Camaraderie	18 (5.4% [3.4-8.4])	18 (14.8 [9.5-22.2])	0
	Positive romantic relationship	79 (23.6% [19.3-28.4)	0	79 (37.1% [30.9-45.9])
Total references – positive themes (references per minute)		335 (0.4 references per minute)	122 (0.3 references per minute)	213 (0.6 references per minute)

The most common adult themes in our sample of 200 songs were “sexually suggestive lyrics” (n=1062, 32.2%), “sexually suggestive dancing” (n=514, 15.6%), “use of swear words” (n=453, 13.7%), “alcohol use” (n=155, 4.7%), and “use of guns/deadly weapons” (n=155, 4.7%). The most common positive theme in our sample of 200 songs was “empowerment” (n=210, 62.3%).

When comparing our two stratified study periods, there was a greater absolute number of total adult (1820 vs. 1478) and positive (213 vs. 122) references in the 2009-2018 study period compared with the 1999-2008 study period. (Table 3) When comparing our two stratified study periods, while there were greater references per minute for adult themes (4.8 vs. 3.7 references per minute) and positive themes (0.6 vs. 0.3 reference per minute) in the 2009-2018 study period compared with the 1999-2008 study period, this was not statistically significant (p=0.38 for adult themes and p=0.41 for positive themes). (Table 3) When comparing our two stratified study periods, there was an increase over time in the total number of major adult themes except for “sexuality” and “partying”. Lastly, when comparing our two stratified study periods, there was an increase over time in the total number of positive themes except for “camaraderie”.

Table 4 demonstrates the total number of references for the adult and positive themes, as well as the references per minute, stratified by each year during the 20 year study period. Variability can be seen in the total number of references and references per minute during the 20-year study period.

**Table 4:** Total number of adult and positive theme references, stratified by each year (1999 to 2018)

				1999		2000		2001		2002		2003		2004		2005		2006		2007		2008		2009		2010		2011		2012		2013		2014		2015		2016		2017		2018		Total		
Violent crime (7.2%)				1		1		3		0		4		5		5		63		0		10		2		13		30		2		0		4		0		10		67		16		236		
		Physical assault (20.3%)		0		0		1		0		2		5		5		6		0		9		0		7		7		2		0		2		0		0		1		1		48		
		Sexual assault 0%		0		0		0		0		0		0		0		0		0		0		0		0		0		0		0		0		0		0		0		0		0		
		Murder (3.4%)		0		0		0		0		0		0		0		0		0		0		0		0		0		0		0		0		0		8		0		0		8		
		Self-harm (10.6%)		0		0		0		0		1		0		0		0		0		0		0		0		23		0		0		0		0		0		1		0		25		
		Use of guns/deadly weapons (65.7%)		1		1		2		0		1		0		0		57		0		1		2		6		0		0		0		2		0		2		65		15		155		
Non-violent crime (4.5%)				1		2		0		2		1		9		1		3		12		0		49		4		1		10		5		1		0		12		15		19		147		
		Theft (22.4%)		1		2		0		2		0		3		0		0		0		0		0		0		0		1		3		0		0		12		9		0		33		
		Gambling (30%)		0		0		0		0		0		0		0		0		0		0		44		0		0		0		0		0		0		0		0		0		44		
		Glorification of prison (3.4%)		0		0		0		0		0		1		0		1		0		0		0		0		1		0		2		0		0		0		0		0		5		
		Gang glorification (23.1%)		0		0		0		0		0		5		1		2		0		0		0		2		0		1		0		0		0		0		6		17		34		
		Vandalism (21.1%)		0		0		0		0		1		0		0		0		12		0		5		2		0		8		0		1		0		0		0		2		31		
Use of inappropriate language (20%)				11		9		19		11		38		37		54		25		12		10		42		15		40		21		43		7		33		34		136		64		661		
		Use of swear words (68.5%)		2		7		16		9		16		14		52		13		8		5		42		12		36		21		41		6		31		16		61		45		453		
		Racism (13.3%)		0		1		3		1		10		19		2		7		1		5		0		0		2		0		0		0		1		8		25		3		88		
		Homophobia (0.6%)		0		0		0		0		2		1		0		0		0		0		0		0		0		0		0		0		0		0		1		0		4		
		Sexual harassment (17.6%)		9		1		0		1		10		3		0		5		3		0		0		3		2		0		2		1		1		10		49		16		116		
Sexuality (36.4%)				41		35		36		56		110		67		76		62		65		124		36		22		94		5		47		58		56		16		153		43		1202		
		Sexually suggestive lyrics (88.3%)		32		6		36		36		102		47		76		62		56		118		36		18		93		3		27		58		55		16		143		42		1062		
		Performing sexual acts (6.4%)		0		18		0		7		7		3		0		0		1		6		0		4		1		2		18		0		1		0		9		0		77		
		Infidelity (5.0%)		9		11		0		13		0		17		0		0		8		0		0		0		0		0		0		0		0		0		1		1		60		
		Prostitution (0.3%)		0		0		0		0		1		0		0		0		0		0		0		0		0		0		2		0		0		0		0		0		3		
Substance use (10.7%)				2		0		1		4		20		3		5		13		22		13		10		16		33		17		7		12		42		23		84		27		354		
		Alcohol use (43.8%)		0		0		1		4		11		2		3		8		18		12		10		13		26		15		2		7		3		8		7		5		155		
		Selling drugs (22%)		0		0		0		0		0		0		1		0		2		0		0		0		1		0		0		0		14		8		51		1		78		
		Drug use (34.2%)		2		0		0		0		9		1		1		5		2		1		0		3		6		2		5		5		25		7		26		21		121		
Partying (21.2%)				1		0		15		2		41		104		22		31		14		144		58		40		29		7		12		2		49		90		27		10		698		
		Sexually suggestive dancing (73.6%)		1		0		8		0		30		57		19		27		6		100		52		17		20		7		7		2		44		87		22		8		514		
		Partying/clubbing (26.4%)		0		0		7		2		11		47		3		4		8		44		6		23		9		0		5		0		5		3		5		2		184		
Total references -adult themes				57		47		74		75		214		225		163		197		125		301		197		110		227		62		114		84		180		185		482		179		3298		
Number of references per minute - Adult themes				1.4		1.1		1.7		1.8		5.3		5.6		4.5		5.1		3.1		7.1		5.0		2.8		6.0		1.7		2.7		2.4		4.6		4.9		12.8		4.7		4.2		
Total references - positive themes					36		0		28		0		0		0		0		39		15		4		0		0		23		40		13		106		4		0		23		4		335	
Number of references per minute - positive themes					0.9		0		0.7		0		0		0		0		1.0		0.4		0.1		0		0		0.6		1.1		0.3		3.0		0.1		0		0.6		0.1		0.4	
			Positive body image (8.3%)		0		0		0		0		0		0		0		9		2		0		0		0		0		0		0		13		1		0		3		0		28	
			Empowerment (62.7%)		31		0		28		0		0		0		0		30		0		4		0		0		23		24		13		53		2		0		2		0		210	
			Camaraderie (5.4%)		5		0		0		0		0		0		0		0		13		0		0		0		0		0		0		0		0		0		0		0		18	
			Positive romantic relationship (23.6%)		0		0		0		0		0		0		0		0		0		0		0		0		0		16		0		40		1		0		18		4		79	

## Discussion

Based on our sample of contemporary popular music, there were more adult theme references than positive theme references. The most common adult themes in our sample were “sexually suggestive lyrics,” “sexually suggestive dancing,” “use of swear words,” “alcohol use,” and “use of guns/deadly weapons.” The most common positive theme in our sample was “empowerment.” When comparing our two stratified study periods, there was an increase over time in the total number of adult themes except for “sexuality” and “partying.” Lastly, when comparing our two stratified study periods, there was an increase over time in the total number of positive themes except for “camaraderie.”

Although there have been several other studies examining the lyrical content of music, our study focuses on a variety of genres over two decades (1999-2018), focusing on both adult themes and positive themes. Several studies have focused on alcohol and drug use content. One study analyzing 279 popular songs in 2005 from several genres, focusing on substance use, determined that adolescents were exposed to 35.2 substance references per song-hour, with tobacco and alcohol use being the most common references [21]. A content analysis study examining alcohol references in 409 rap songs (1979-2009) found significant increases in alcohol references during their study period, with increases in the association of alcohol with glamor and wealth, drugs, and nightclubs [22]. Another study examining substance use references in 508 Australian top 20 songs (1990-2015) found an increase in references over the study period [23]. Lastly, a study examining 1200 top 40’s songs (1986-2016) found increasing references to opioids, marijuana, and alcohol over the study period [24].

There have also been studies examining the lyrical content of music, specifically references to violence and sexual behavior. A study examining violence in 340 rap songs (1979-1997) determined that there was an increase in references to violence during the study period, depicted in a positive light by its association with glamor, wealth, masculinity, and personal prowess [25]. One study examining the risk behavior content of 600 top 20 songs (2009-2013) found that 35% of the songs mentioned sexual behaviors and that an association with alcohol and a disregard for consequences was common [26]. Lastly, a study of 279 popular songs in 2005, focusing on degrading and non-degrading sexual references, determined that 37% of songs contained references to sexual activity, often associated with references to substance use, violence, and weapon carrying [27]. Questions on whether children and adolescents who are exposed to music actually listen to and interpret the lyrics have been confirmed by published studies [28].

It has been suggested that pediatric healthcare providers take responsibility in the media education of children and adolescents. The American Academy of Pediatrics’ Council on Communications and Media suggest the following responsibilities: 1) become familiar with the content of contemporary popular music and with the public health risks of music on children and adolescents, 2) include media-related questions at each well-child visit to gauge level of exposure and potential risk factors while promoting healthy media habits, 3) encourage parents/guardians to take an active role in monitoring the types of music and in regulating the duration of music exposure based on the age and emotional/development stage of their child or adolescent, 4) participate in local and national coalitions, including schools, to discuss the effects of music on children and adolescents, 5) encourage stakeholders in the music industry to create and release music promoting positive themes, such as developing healthy relationships, eliminating discrimination, alcohol and drug avoidance, nonviolent conflict resolution, sexual abstinence, pregnancy prevention, and healthy body images [1,19]. Future studies should focus on the level of involvement of pediatric healthcare providers in promoting the media education of children and adolescents and whether this education affects the overall wellbeing and social development of children and adolescents who are exposed to music.

We have identified several limitations. Since our analysis was performed on only 200 songs identified by Billboard, with the majority of the songs categorized as Pop, R&B/Soul, and Hip Hop/Rap, our conclusions may not be generalizable to music outside our study period, less popular songs on the music charts during our study period, or less represented genres such as Rock, Electronic, Techno, Jazz, Blues, Folk, Country, Alternative/Indie, Reggae, House, Heavy Metal, Musical theatre, or Christian/Gospel. Secondly, although our objective was to perform a content and trend analysis of contemporary popular music between 1999 and 2018, with particular attention to adult themes and positive themes, we did not examine whether the lyrical content of music in our sample affected pediatric listeners. Despite this limitation, there have been several published studies showing that exposure to music can affect behavior and emotion [8] and can increase risk-taking behaviors, such as aggression/violence [9-10], suicide [11-13], use of tobacco, alcohol, and illicit drugs [14-16], and unsafe sexual practices [17]. Lastly, data collection for each song in our sample was performed as a group; interpretation of lyrical content may be dependent on each individual’s emotional or developmental stage, background or previous experiences, or demographics, such as age, sex, race, or ethnicity. In order to account for these potential differences, our study design should have included an independent completion of the data collection instrument for each song by multiple reviewers from a diverse background, and discrepancies discussed collectively, with final decisions on the categorization of references based on these discussions.

## Conclusions

Based on our sample of contemporary popular music, there were more adult theme references than positive theme references, with the most common adult themes related to sexual behavior and alcohol use, and the most common positive theme related to empowerment. Furthermore, when comparing our two stratified study periods, there was an increase over time in the total number of major adult themes except for “sexuality” and “partying,” and there was an increase over time in the total number of positive themes except for “camaraderie.”

We encourage pediatric healthcare providers to be active participants in the promotion of media education as delineated by the American Academy of Pediatrics. We encourage parents and guardians to be mindful of the level of media exposure of their children and adolescents. We encourage the music industry to create music appropriate for children and adolescents that focuses on positive themes such as developing healthy relationships, eliminating discrimination, alcohol and drug avoidance, nonviolent conflict resolution, sexual abstinence, pregnancy prevention, and healthy body images.
